# Dispersal Enhances Abiotic Adaptation in Populations Under Interspecific Competition Compared to Isolation

**DOI:** 10.1002/ece3.73358

**Published:** 2026-04-08

**Authors:** Dong‐Hao Zhou, Shu‐Jun Chang, Nan Chen

**Affiliations:** ^1^ College of Ecology and Environment (College of Wetlands) Southwest Forestry University Kunming Yunnan China; ^2^ National Plateau Wetlands Research Center Kunming Yunnan China; ^3^ State Key Laboratory of Earth Surface Processes and Resource Ecology and MOE Key Laboratory for Biodiversity Science and Ecological Engineering, College of Life Sciences Beijing Normal University Beijing China

**Keywords:** among‐habitat dispersal, evolutionary adaptation, experimental evolution, interspecific competition, temperature

## Abstract

Interspecific competition is known to hinder evolutionary adaptation by limiting resource availability and reducing genetic diversity. In contrast, dispersal has been hypothesized to mitigate these limitations by increasing genetic variation and promoting gene flow. Here, we investigated how dispersal influences abiotic adaptation in the presence and absence of interspecific competition using two bacterial species, *Escherichia coli* and *Pseudomonas fluorescens* . Populations were cultured under monoculture and coculture conditions across three temperatures, with and without dispersal. Our results revealed that dispersal significantly enhanced abiotic adaptation in populations in competitive environments, particularly in numerically inferior populations, facilitating persistence under warmer conditions. Dispersal also prevented extinction events observed in cocultures without dispersal, highlighting its role in population persistence. These findings underscore the importance of adaptation to global warming, particularly for inferior species.

## Graphical abstract

Each circle represents an individual with a different hue indicating differences in species, while the shade of the color reflects the degree of abiotic adaptation. Competition may hinder abiotic adaptation of inferior populations, but dispersal mitigates such consequences.

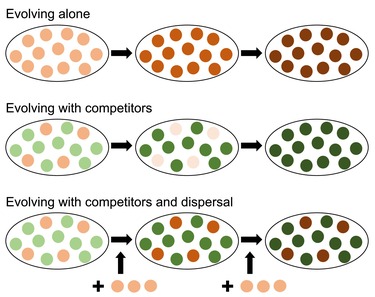

## 1 Introduction

Competition is a cornerstone of Darwinian theory, shaping speciation, extinction, and community dynamics (Darwin 1859; Hall et al. 2018). Dispersal, another fundamental ecological process, facilitates genetic exchange, connects spatially isolated populations, and influences community assembly and turnover (Berdahl et al. 2015; Garant et al. 2007). The interplay between competition and dispersal has profound implications, extending beyond ecology to evolutionary biology, epidemiology, and conservation (Girardin 2019).

Competition is believed to promote character displacement, niche differentiation, and species divergence, and thus may accelerate evolutionary adaptation (Arthur 1982; Brown and Wilson 1956; Dal Bello et al. 2021; Darwin 1859). However, the ecological opportunities required for these outcomes are not always present (Kassen 2014; Wellborn and Langerhans 2015). In many cases, competition restricts resource access for focal populations, reducing population size (Gause and Witt 1935). This decline in population size diminishes genetic diversity and amplifies the effects of genetic drift relative to natural selection, thereby constraining abiotic adaptation (Johansson 2008; Osmond and de Mazancourt 2013; Vahdati et al. 2017). Additionally, species in competitive environments often exhibit narrower ecological niches, further limiting the availability of beneficial mutations required for adaptation (Chen and Zhang 2024; Sale 1974). These factors collectively contribute to species extinction under competitive pressure (Johansson 2008).

In contrast, dispersal serves as a mechanism for population persistence, primarily by accelerating gene flow (Berdahl et al. 2015; Freeland 2020; Garant et al. 2007). Dispersal allows maladapted individuals to colonize new habitats, reducing extinction risk and increasing the proportion of high‐fitness genotypes in native populations. Furthermore, intraspecific genetic variation directly benefits adaptation through mechanisms such as enhanced group performance, stability, resistance to invasion, and effective responses to natural selection (Agashe 2009; Lande and Shannon 1996; Spong et al. 2000). Note that excessively high gene flow may incur genetic load or genetic homogenization which impedes abiotic adaptation in populations (Alzate and Hagen 2024; Duncan et al. 2015).

Dispersal introduces migrants from alternative environments, which can have two significant effects on native populations: increased effective population size (*N*_e_) and enhanced genetic variation. The former results from the addition of individuals, while the latter arises from genetic mixing due to local adaptation in alternative habitats (Garant et al. 2007). A larger and stable *N*_e_ minimizes the impact of genetic drift, facilitates the fixation of beneficial mutations, maintains mating opportunities, and reduces inbreeding depression (Berdahl et al. 2015; Ellstrand and Elam 1993; Freeland 2020; Pinto et al. 2023). Populations with greater genetic diversity are better equipped to adapt to environmental changes and adverse conditions (Agashe 2009; Lande and Shannon 1996; Spong et al. 2000). According to these ecological consequences, the effects of dispersal on abiotic adaptation in populations in competitive environments are particularly significant for four reasons. First, competitive abilities often depend on inherent resource‐uptake traits, which are difficult to improve over short ecological timescales and can only be modified through long‐term evolutionary processes (Goldberg 1990). Second, populations in competitive environments typically face resource scarcity, hindering their ability to invest in evolutionary innovations such as utilizing alternative resources. Limited resources and energy are primarily allocated to acquiring critical resources (Bisschop et al. 2020; Blossey and Notzold 1995; Collins 2011). Third, populations in competitive environments generally have smaller *N*_e_ and lower genetic diversity, making dispersal more effective in increasing both metrics. Together, these mechanisms make dispersed individuals crucial for significantly enhancing *N*_e_, genetic diversity, and thus fitness of abiotic adaptation. Last, interspecific competition per se exerts a stronger selection pressure on populations (Alzate et al. 2017; Osmond and de Mazancourt 2013), for example, decreasing the availability of resources, causing maladaptive alleles from dispersed populations to be eliminated while beneficial alleles spread effectively. These dynamics make beneficial mutations introduced through dispersal more impactful on population fitness than spontaneous mutations fixed in native populations. Notably, the effects of dispersal vary depending on a population's dominance. In dominant populations, the impact of dispersal on adaptation is expected to be relatively limited. Such populations have occupied sufficient resources, enabling investment in evolutionary innovations, and possess larger *N*_e_ and more standing genetic variations. Consequently, immigrant individuals may less significantly increase *N*_e_ or genetic diversity, resulting in minor improvement in the rate of evolutionary adaptation to abiotic conditions; in contrast, inferior populations, which possess smaller population sizes, are likely to derive greater benefits from dispersal (Chen and Zhang 2024; Gause and Witt 1935; Osmond and de Mazancourt 2013).

To determine whether the effects of dispersal are more pronounced in antagonistically interacting species compared to populations evolving in isolation, we conducted a long‐term experimental evolution study using two model bacterial species, *Escherichia coli* and *Pseudomonas fluorescens* . Populations were grown under monoculture and coculture conditions, with and without dispersal. These experimental setups were maintained across three temperatures for 2 years (approximately 2400 generations), after which the abiotic adaptation of each population was assessed and compared. The present study is a follow up on a previous study (Chen and Zhang 2024).

## 2 Material and Methods

### 2.1 Stains and Culture Conditions

The evolution experiment used *E. coli* strain B REL606 (Siegel et al. 1982) and *P. fluorescens* strain SBW25EeZY6KX (Bailey et al. 1995), both strictly asexual. Notably, the latter strain exhibits resistance to the antibiotic kanamycin. The bacterial populations were grown statically in 50 mL centrifuge tubes with loosely sealed lids in incubators, and each tube contained 5 mL of 0.1 × M9KB liquid medium that consisted of M9 buffer, 2 g L^−1^ of proteose peptone No. 3 and 1 g L^−1^ of glycerol. Throughout the experiment, we carried out a serial batch culture approach with 100‐fold dilution every 2 days. Specifically, 50 μL of each culture was transferred into 4.95 mL of fresh medium. Under these specific culture conditions, *E. coli* could maintain viable across 15°C–41°C and be dominant at warmer temperatures (i.e., excluding *P. fluorescens* at temperatures > 31.5°C), while *P. fluorescens* exhibited a temperature tolerance across 6°C–34°C and was dominant at colder temperatures (i.e., excluding *E. coli* at temperatures < 18°C) (Chen and Zhang 2023).

### 2.2 The Evolution Experiment

*E. coli* and *P. fluorescens* monocultures, and their cocultures were incubated at three temperatures, 19°C, 26°C and 31°C. Each kind of culture included two lines, with and without among‐habitat dispersal. Nine incubators were employed and randomly organized into three distinct blocks. Within each block, the three incubators were maintained at 19°C, 26°C, and 31°C, respectively. Two replicates were established within each incubator. Each replicate included six microcosms: *E. coli* monocultures, *P. fluorescens* monocultures and cocultures, and the rest were same as the three cultures above but with among‐temperature dispersal (Figure S1a). The forced dispersal happened between same kinds of cultures at different temperatures (incubators) within a block and same replicate ID (Figure S1b). Each culture was initially inoculated with approximately 10^7^ bacterial cells and with the ratio of 1:1 of two species for coculture. The evolution lines subsequently underwent the 100‐fold dilution at 2‐day intervals, and such dilution process was different between cultures with and without dispersal. Specifically, for those without dispersal, original samples transferred into fresh medium were all from previous cultures with same ID; while for those with among‐habitat dispersal, 90% (45 μL) original samples were from previous cultures with same ID, and the rest 10% (5 μL) were from mixed previous cultures at different temperatures within a block and same replicate and microcosm ID. Noteworthily, each culture consistently achieved a stationary growth phase during each round of population expansion, and 6.64 generations of binary fission occurred within each transfer in monocultures (Kassen 2014). Every 200 generations (30 days), samples of evolved strains from each culture were collected and frozen at −80°C with glycerol (final glycerol content: 30%). We also performed contamination checking in each stock step and the previously frozen‐stored samples would be recovered and used if contamination occurred in certain lines.

Population densities, expressed as colony forming units per mL (CFUs mL^−1^), were measured at generation 27, 100, 200, and then every 200 generations. Density measurements for the two pure species were obtained using distinct selective agar plate cultivation conditions. Specifically, densities of *E. coli* populations were determined by diluting the culture and plating it on M9KB agar, followed by incubation at 37°C for 1 day, a temperature where *P. fluorescens* could not grow. Densities of *P. fluorescens* were determined using M9KB agar plates supplemented with 100 mg L^−1^ of kanamycin, followed by incubation at 26°C for 2 to 3 days, where *E. coli* was unable to grow. It is noteworthy that a small fraction of evolved *E. coli* strains may obtain kanamycin resistance due to spontaneous mutations, potentially leading to mismeasurements of *P. fluorescens* , particularly when *P. fluorescens* is extremely rare. In such situations, the susceptibility of colonies was checked through exposure to phage T3 (that could infect *E. coli* ) (Rodríguez‐Verdugo et al. 2014) and phage SBW25Φ2 (that could infect *P. fluorescens* ) (Buckling and Rainey 2002), respectively. Specifically, the colonies to be examined were picked and transferred to the fresh liquid medium on 96‐well microplates for growth for 2 days at 26°C. The grown samples were then transferred into another fresh medium with no phage, with T3, or with SBW25Φ2, respectively on 96‐well microplates for growth for 8 h at 26°C. Cultures that could not grow with T3 were identified as *E. coli* and those that could not grow with SBW25Φ2 were *P. fluorescens* . We did not observe any evolving *P. fluorescens* population which could form colonies at 37°C. Note that this verification method confirmed the extinction of *P. fluorescens* in four cocultured lines without dispersal at 31°C over the course of the evolution experiment.

We used two selective liquid incubation environments to isolate the single‐species populations from cocultured lines at generation 2400. Specifically, the isolation of *E. coli* populations was achieved by transferring 5 μL of cocultures into 5 mL of fresh medium, which was subsequently grown at 40°C for 2 days. The isolation of *P. fluorescens* was performed by transferring 5 μL of cocultures into 5 mL of fresh medium with 100 mg L^−1^ of kanamycin, which was subsequently grown at 10°C for 2 days. To confirm the success of isolation, 2 μL of samples from the heat‐treated and antibiotic‐treated cultures above were transferred into 200 μL of fresh medium with and without kanamycin in 96‐well microplates respectively, and were incubated at 10°C and 40°C respectively. Each isolated sample was carried out three replicate assays, and the turbidity of these cultures in 96‐well after 2‐day growth suggested the possibility of failure of isolation. To further confirm the purity of isolated populations, we measured densities of both target species and their previous competing species after growing focal populations for more than four transfers. Success of population isolation was ensured in all cocultured lines.

### 2.3 Fitness Assay

Samples stocked at generation 2400 (transfer 360) were studied here. Both monocultures and cocultures underwent bacterial isolation procedures (see above) before fitness assays. Relative fitness of each population and their ancestral strains was estimated in the absence of interspecific competition (Collins 2011; Gómez et al. 2022; Gorter et al. 2021; Hall et al. 2018), at all the three experimental temperatures, 19°C, 26°C, and 31°C, through head‐to‐head competition assays (Lenski et al. 1991). Such value estimates the adaptation to abiotic environments. We assessed the fitness of evolved *E. coli* populations and their ancestral strain (all of the strain REL606) in comparison to a reference strain, REL606Ara+. Colonies of REL606 and REL606Ara+ showed red and white colors on TA agar plates, respectively. Note that a small subset of the evolved REL606 populations in our experiment exhibited colonies with nearly white colors. But they could be distinguished from the reference strain based on absolutely different colony morphology. For evolved *P. fluorescens* and their ancestor (all of the strain SBW25EeZY6KX), SBW25 was chosen as a reference strain. On M9KB agar plates with X‐gal, colonies of SBW25EeZY6KX showed blue color and SBW25 showed yellow color.

Measurement of fitness involved three steps: reconditioning, acclimation and competition assays. In reconditioning step, 5 μL of tested and reference frozen cultures were inoculated into 5 mL of nutrient medium respectively and grown for 2 days, *E. coli* at 37°C and *P. fluorescens* at 28°C. In acclimation step, 5 μL of reconditioned cultures were transferred into fresh medium and grown for 2 days at evolution temperatures. In competition assays step, 25 μL of the acclimated tested population and 25 μL of its reference strain were added together into 5 mL of fresh medium and incubated for 2 days at evolution temperatures. Densities of the two strains were measured by plating culture dilutions onto indicator plates (TA agar plates for *E. coli* ; M9KB agar plates with X‐gal for *P. fluorescens* ) immediately after inoculation and at the end of incubation. Relative fitness of each tested population against the reference strain was calculated as the ratio of Malthusian parameters, *W* = *m*_tested_/*m*_reference_, *m* = ln (*N*_f_/*N*_0_), where *N*_0_ and *N*_f_ are the initial and final densities, respectively.

Change in fitness in a certain population against its ancestor was calculated as the difference between the two: *W*_evolved_ − *W*_ancestor_, which is akin to the selection coefficient index (Lenski et al. 1991; Lopez‐Pascua and Buckling 2008). Such index was dependent on assay environments and thus was not comparable across assay temperatures.

### 2.4 Impact of Dispersal

An index of the effect of dispersal on abiotic adaptation was calculated as ln (*W*_dispersal_/*W*_isolation_), where *W*_dispersal_ and *W*_isolation_ are the relative fitness of the identical population in the same incubator (A, B or C), replicate (a or b) and culture type (monoculture or coculture) with and without dispersal respectively. We estimated such index for populations of each species from monocultures and cocultures at each experimental temperature. This estimate for “effects of dispersal” was comparable across assay environments; and positive values suggested faster evolutionary adaptation caused by dispersal.

### 2.5 Statistical Analysis

Data analyses were performed using R 4.0.2 (R Core Team 2020). One‐sample *t*‐test was used to analyze whether dispersal enhanced abiotic adaptation, that is, the value of ln (*W*_dispersal_/*W*_isolation_) is more than 0. Paired *t*‐test was used to analyze the difference in “effects of dispersal” between evolution and alternative environments, and was also used to analyze the difference in change in fitness of populations evolving in monocultures versus cocultures without and with dispersal; Benjamini‐Hochberg method was used to adjust *p*‐values for non‐independent multiple comparisons. Note that no statistical test was performed for *P. fluorescens* from 31°C cocultures without dispersal due to the extremely small sample size (*n* = 2).

## 3 Result

### 3.1 Effects of Dispersal on Populations Evolving Alone

**FIGURE 1:**
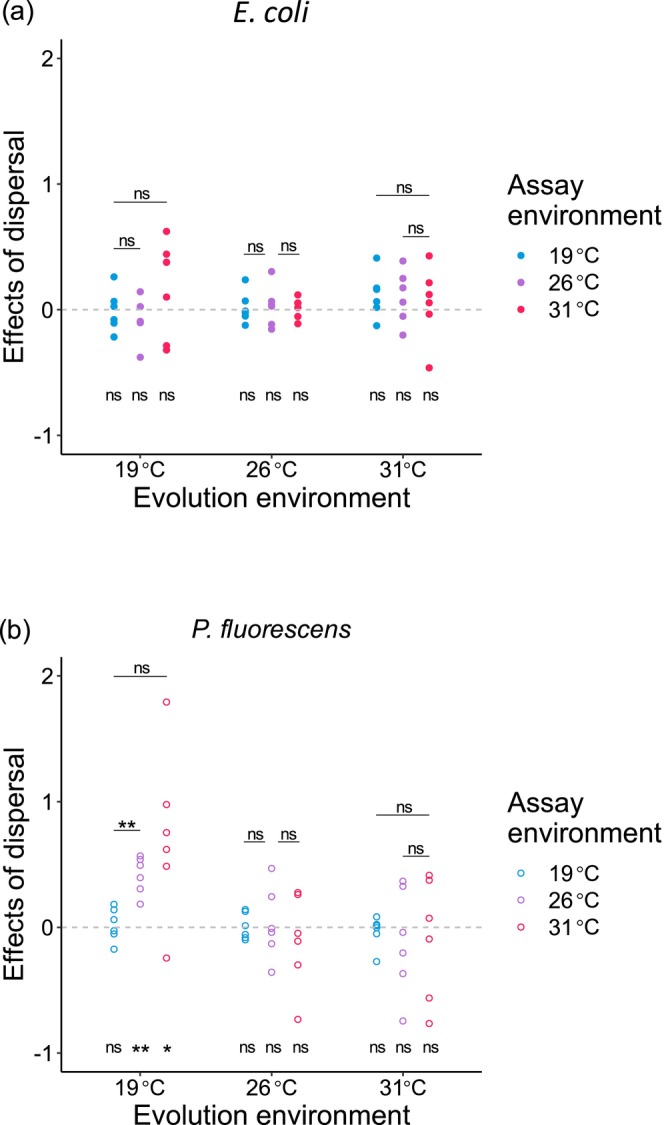
Effects of among‐population dispersal on abiotic adaptation (log‐transformed ratio of relative fitness of populations with dispersal to those without) of monocultured populations assayed at all the three temperatures. Separate analyses were performed for populations of the two species (a) *E. coli* and (b) *P. fluorescens* . Significant differences between effects of dispersal and the expected value of zero, and the effects in experimental evolution environment and in alternative environments were indicated by asterisks (based on paired *t* tests, *p < 0.05; **p < 0.01; ***p < 0.001; ns, non‐significant).

We first examined the strength of dispersal effects on abiotic adaptation in those monocultured populations. Results of this part showed little difference between two model species (Figure 1). Specifically, for *E. coli* populations that evolved at each temperature, the effects of dispersal did not significantly increase fitness in both evolution and alternative environments (Figure 1a). In addition, the effects of dispersal did not vary in adaptation to evolution and alternative environments (Figure 1a).

For *P. fluorescens* populations evolving at 19°C, immigrants from warmer monocultures significantly enhanced adaptation to two alternative environments, and the effects of dispersal for these evolution lines on accelerating adaptation to an alternative temperature, 26°C, were stronger than on accelerating adaptation to evolution temperature (Figure 1b). However, for these evolution lines, dispersal among habitats did not significantly increase their fitness at evolution temperature, and their ability for colonization in 31°C environment was not significantly reinforced by immigrants compared to evolution environment (Figure 1b). For *P. fluorescens* lines evolving at 26°C and 31°C, their adaptation to evolution and alternative environments was not significantly improved by dispersal, and the effects of dispersal were the same in accelerating adaptation to evolution and alternative environments (Figure 1b).

### 3.2 Effects of Dispersal on Populations in Competitive Environments

**FIGURE 2:**
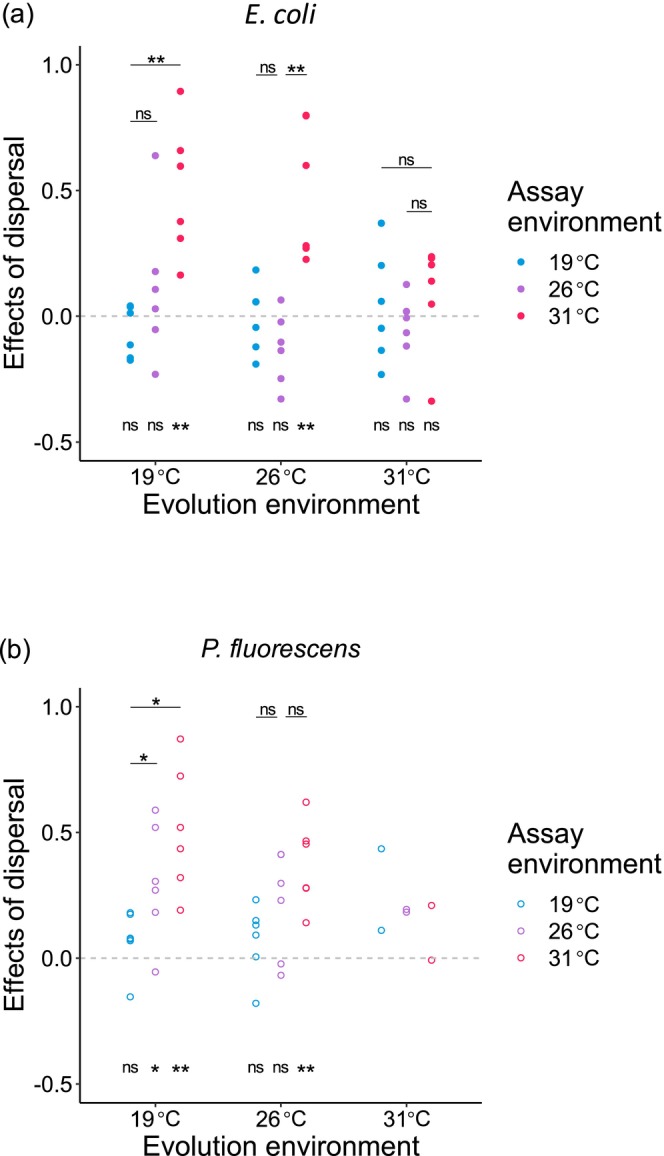
Effects of among‐community dispersal on abiotic adaptation (log‐transformed ratio of relative fitness of populations with dispersal to those without) of cocultured populations assayed at all the three temperatures. Separate analyses were performed for populations of the two species (a) *E. coli* and (b) *P. fluorescens* . Symbols as in Figure 1. Note that no statistical test was performed for *P. fluorescens* from cocultures at 31°C due to the very small sample size of 2 in lines without dispersal.

We then tested how dispersal among communities affected abiotic adaptation. Partly as expected, we found that dispersal accelerated abiotic adaptation in some scenarios. Specifically, for *E. coli* populations from cocultures, both lines evolving at 19°C and 26°C adapted better to 31°C alternative environment when dispersal happened (Figure 2a). And dispersal made such evolution lines increased more fitness at 31°C than at their evolution temperatures (Figure 2a). However, dispersal did not significantly enhance adaptation of 19°C‐origin populations at their evolution temperature and 26°C, and either adaptation of 26°C‐origin lines at their evolution temperature and 19°C (Figure 2a). *coli* evolving at 31°C, immigrant individuals from communities in colder environments did not improve their abiotic adaptation in both evolution and alternative environments (Figure 2a), and the intensity of dispersal effects was not different across the three experimental temperatures (Figure 2a).

**FIGURE 3:**
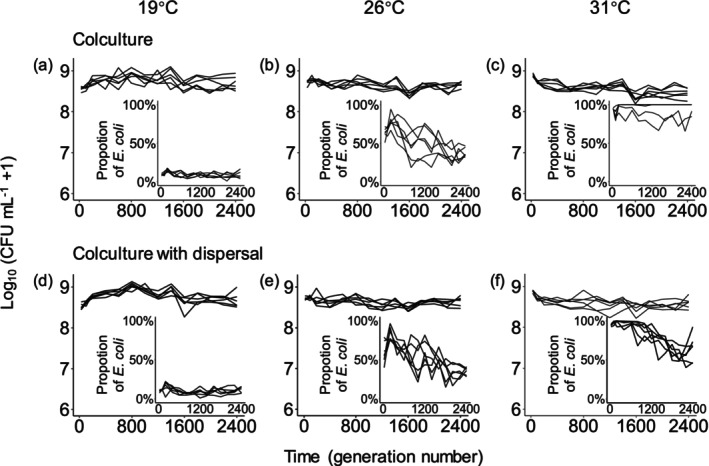
Densities of bacteria in cocultures over time during the evolution experiment. The inserted panels show the proportion of *E. coli* . Each line indicates a single evolution line. Population extinction was observed in four cocultures without dispersal, all in the 31°C environment (*P. fluorescens* extinction). Panels (a) to (c) are from our previous work (Chen and Zhang 2024).

Note that *E. coli* kept numerically inferior at 19°C in all scenarios (average proportion in cocultures without dispersal: 9.4%; average proportion in cocultures with dispersal: 10%; Figure 3a,d), and absolutely and marginally dominant at 31°C in coculture without and with dispersal, respectively (average proportion in cocultures without dispersal: 94%; average proportion in cocultures with dispersal: 81%; Figure 3c,f). In 26°C cocultures, *E. coli* is competitively comparable (average proportion in cocultures without dispersal: 52%; average proportion in cocultures with dispersal: 53%; Figure 3b,e). The significant positive effects of dispersal on adaptation to 31°C and more fitness improvement in adaptation to 31°C than adaptation to evolution temperatures in *E. coli* populations evolving at lower temperatures suggested that being less dominant enhanced the effects of dispersal on abiotic adaptation to new environments.

For *P. fluorescens* populations evolving at 19°C, dispersal significantly increased their fitness at 26°C and 31°C, and the effects of dispersal were significantly greater when adapting to the 26°C environment than to the evolution environment, which were the same as the scenarios without dispersal (Figures 1b and 2b). But these evolution lines showed more fitness elevation in the 31°C environment than in the evolution environment when dispersal happened (Figure 2b). In addition, significant effects of dispersal on change in fitness were found in 26°C‐origin *P. fluorescens* competitive populations when they adapted to the 31°C environment (Figure 2b). Dispersal did not significantly affect abiotic adaptation of *P. fluorescens* populations from 19°C to 26°C cocultures to their evolution environments (Figure 2b). For *P. fluorescens* evolving at 26°C, fitness in 19°C was not significantly different between those with dispersal and those without; and the effects of dispersal did not make such populations improve more in adaptation to alternative environments than to the evolution environment (Figure 2b). *P. fluorescens* from 31°C cocultures with dispersal seemed to have higher fitness compared with those without dispersal at each assay environment (though no statistical test was performed due to the small sample size; Figure 2b).

Notably, the presence of competitor, *E. coli* , did not change the degree of adaptation to all the three temperatures for 19°C‐origin *P. fluorescens* populations which were dominant in competition (average proportion in cocultures without dispersal: 91%; average proportion in cocultures with dispersal: 90%). While competition made the effects of dispersal significant in 26°C‐origin *P. fluorescens* populations which were comparable in competition (average proportion in cocultures without dispersal: 48%; average proportion in cocultures with dispersal: 47%; Figure 3b,e). Unfortunately, most of *P. fluorescens* populations without dispersal went extinct in cocultures before the fitness assay, thus it was unable to explore their abiotic adaptation when they were extremely inferior (Figure 3c).

### 3.3 Effects of Dispersal on Impacts of Competition

**FIGURE 4:**
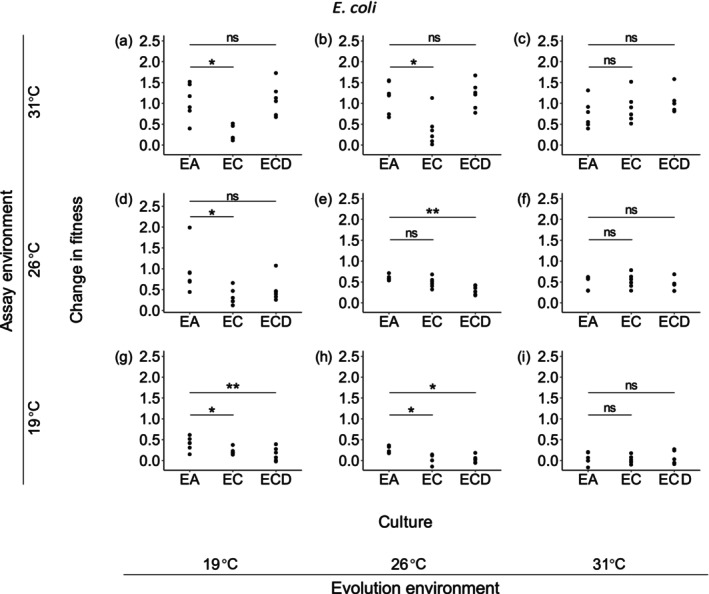
Change in fitness of *E. coli* populations in three kinds of cultures at all the three temperatures. EA, populations evolving alone; EC, evolved populations isolated from cocultures; ECD, evolved populations isolated from cocultures with among‐community dispersal. Each species in each culture type (monoculture or coculture) evolving at three temperatures, 19°C, 26°C, and 31°C, and the fitness of each evolved population was assayed at three temperatures, 19°C, 26°C, and 31°C. Significant differences between monocultured and cocultured populations, and monocultured populations and cocultured populations with dispersal were indicated by asterisks (based on paired *t* tests). ns, non‐significant.

We then compared increased fitness in monocultures, cocultures with and without dispersal to explore the effects of dispersal on competition consequences. For *E. coli* , the presence of competitors always hindered abiotic adaptation to alternative temperatures when being inferior or comparable, but the constraint on adaptation to higher temperatures was alleviated when dispersal happened (Figure 4a,b,d). In the rest scenarios of *E. coli* populations, dispersal did not mitigate or reinforce impacts of competition on abiotic adaptation except for those from 26°C adapting to evolution environments (Figure 4c,e–i). In such cases, interspecific competition did not impede their abiotic adaptation; rather, the combined effects of competition and dispersal significantly impaired adaptation (Figure 4e).

**FIGURE 5:**
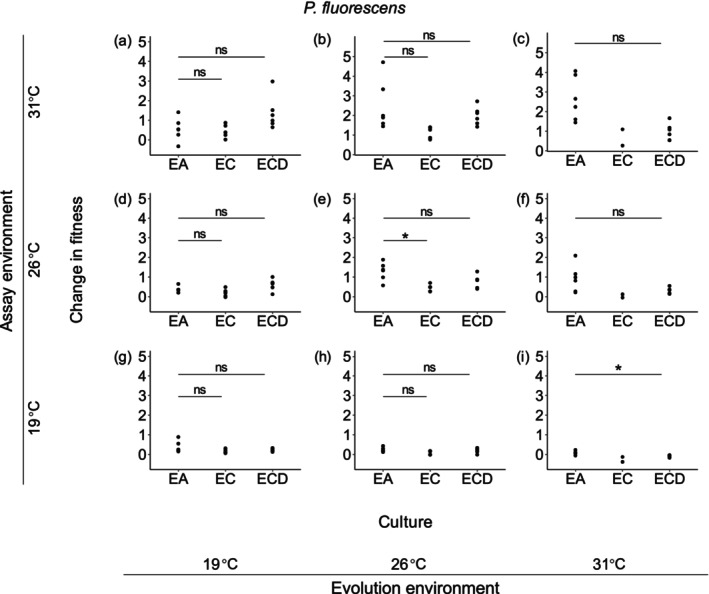
Change in fitness of *P. fluorescens* populations in three kinds of cultures at all the three temperatures. Abbreviations and symbols as in Figure 4. Note that no statistical test was performed for cocultured *P. fluorescens* without dispersal at 31°C due to the very small sample size of 2.

For *P. fluorescens* populations, only decreased abiotic adaptation of 26°C‐origin populations in the evolution environment was observed, which could be obviously mitigated by dispersal (Figure 5e). In the rest of the scenarios of *P. fluorescens* populations, the occurrence of dispersal did not affect the impacts of competition on abiotic adaptation (Figure 5).

## 4 Discussion

Interspecific competition consistently impedes abiotic adaptation, whereas dispersal generally facilitates it (Berdahl et al. 2015; Chu et al. 2021; Freeland 2020; Garant et al. 2007; Hall et al. 2018; Howells et al. 2025; Johansson 2008; Osmond and de Mazancourt 2013). However, their combined effects remain poorly understood. In the present study, we compared the effects of dispersal on populations evolving in isolation and those undergoing interspecific competition to investigate these complex interactions. We found that the effects of dispersal on accelerating abiotic adaptation were more pronounced in the presence of interspecific competitors, particularly for numerically inferior populations. This effect was most evident in populations originating from low‐temperature environments where dispersal facilitated adaptation to higher temperatures and mitigated the negative impacts of competition on abiotic adaptation. Particularly, extinction events were observed in *P. fluorescens* populations within four cocultured lines without dispersal. This outcome was attributed to the superior competitive abilities of *E. coli* , which benefited from faster abiotic adaptation (Chen and Zhang 2024). In contrast, both species persisted in cocultured lines where dispersal occurred. The maintenance of *P. fluorescens* populations in “black‐hole sink” habitats highlights the positive role of dispersal in promoting abiotic adaptation and population persistence under competitive pressure (Garant et al. 2007; Gomulkiewicz et al. 1999).

Our results showed that dispersal was more effective in improving abiotic adaptation in cocultured microcosms compared to monocultures, consistent with the expectation that populations experiencing interspecific competition—characterized by smaller *N*_e_, lower genetic diversity, and limited resource availability—benefit more from dispersal. Note that most dispersal happening in natural communities is spontaneous, suggesting that beyond the significant increase in individual and genetic variation in focal populations, there are other mechanisms caused by dispersal that can promote abiotic adaptation of populations in competitive environments. For example, dispersal can remove maladaptive individuals, reducing population density and competition intensity while increasing the average fitness of the remaining individuals (Branch 1975; Freedberg et al. 2021).

As anticipated, the effects of dispersal were stronger in numerically inferior populations. For both species, adaptation to 31°C improved substantially when populations were inferior or comparable ( *E. coli* in 19°C and 26°C; *P. fluorescens* in 26°C). In contrast, dominant populations ( *E. coli* in 31°C; *P. fluorescens* in 19°C) showed less improvement (Figures 1 and 2). The persistence and higher relative abundance of *P. fluorescens* populations in 31°C cocultures with dispersal, compared to those without dispersal, underscore the importance of sustained migration for inferior species persistence. In natural communities, dominant species often create sink habitats for inferior species which may go extinction if there is no continuous immigration (Johansson 2008; Long et al. 2007). Continuous immigration, whether spontaneous or forced, maintains populations of inferior competitors in the black‐hole sinks, and increases equilibria abundance and abiotic adaptation of endangered species (Gomulkiewicz et al. 1999; Long et al. 2007). The positive feedback between relative abundance and abiotic adaptation in populations under interspecific competition can result in a “Matthew effect” in ecology—common species become even more common, while rare species become rarer. Immigration into numerically inferior populations may counteract this feedback by maintaining their relative abundance, thereby slowing the rate of adaptive evolution in dominant populations relative to inferior ones. When both dominant and inferior populations exhibit comparable capacities for abiotic adaptation, their coexistence is promoted. This mechanism, however, may also result in negative ecological consequences, for example, continuous immigration may enable invasive species to establish populations in novel habitats, potentially causing irreversible damage to ecosystems (Mack et al. 2000). This highlights the importance of obstructing the migration pathways of focal organisms in effectively controlling invasive alien species. Note that whether the conclusion from the present study can be directly expanded to macroecological contexts still need further exploration due to the significant difference between microcosms and natural habitats.

Another intriguing finding was that dispersal enhanced adaptation to the warmest experimental temperature, 31°C, in populations originating from lower temperatures. In contrast, populations evolving in warm environments showed no significant improvements in adaptation to lower temperatures when receiving dispersed communities (Figure 2). Such directional asymmetry may result from the temperature dependence of fitness effects of mutations. Specifically, warmer temperatures accelerate biochemical and biophysical processes and thus reduce the constraints on physiological functions (Arrhenius 1889; Hochachka and Somero 2002), which may enhance the fitness effects of some beneficial mutations and make some mutations with pleiotropic effects show beneficial net fitness effects; therefore, warm temperatures may allow beneficial mutations to show stronger fitness effects and increase the proportion of conditionally beneficial mutations (Chen and Zhang 2023; Chu et al. 2020). Populations evolving in warm environments would fix with more beneficial mutations which confer larger fitness benefits and more conditionally beneficial mutations which show local adaptation to warm environments, and vice versa. Consequently, immigrants from warm environments would help populations in cold environments adapt to warm environments better, whereas those from cold environments could not help populations in warm environments adapt to cold environments better.

Populations competing interspecifically often occupy limited niche space (Sale 1974), particularly for inferior species (Biedma et al. 2020; Jacob et al. 2018). Additionally, warm‐adapted species tend to evolve narrower thermal niches (Payne and Smith 2017). These two factors—restricted niche space and thermal specialization—reduce the availability of unconditionally beneficial mutations. For instance, populations with broad niches can exploit a wider range of resources (Roughgarden 1972), enabling fitness improvement through mutations that enhance the uptake of diverse resources. This limitation in beneficial mutations likely explains why cocultured *E. coli* populations evolving in cold environments exhibited lower adaptability to alternative environments compared to their monocultured counterparts (Figure 3). However, the introduction of dispersed populations may mitigate this limitation in warm environments. Specifically, *E. coli* populations in dispersed communities were primarily derived from warm cultures, which typically evolve faster (Chen and Zhang 2023; Chu et al. 2020) and carry a larger repertoire of high‐temperature‐adapted mutations. Consequently, *E. coli* populations in cold cocultures with dispersal had acquired pre‐adaptive genes for higher temperatures, significantly enhancing their fitness in warm alternative environments. In contrast, though cocultured *E. coli* populations in warm environments also received individuals from colder environments, these dispersed populations contained fewer individuals and exhibited lower genetic diversity in cold‐adaptive traits. Consequently, their contribution to enhancing abiotic adaptation in native populations under cold conditions was minimal. This asymmetry in gene flow underscores the importance of relative abundance in determining the effectiveness of dispersal under interspecific competition.

Interestingly, we also observed a case in which dispersal exacerbated the impacts of competition (Figure 3e). One possible explanation is that dispersal may be accompanied with migration load. Although migration facilitates gene flow and introduces novel genetic material, promoting adaptation (Alzate et al. 2017; Berdahl et al. 2015; Freeland 2020; Garant et al. 2007), local adaptation often renders mutations beneficial in one environment but deleterious or neutral in others (Garant et al. 2007). When a large influx of maladaptive alleles occurs, the fitness of the local population may decrease due to the dilution of beneficial alleles (Alzate et al. 2017; Bolnick and Nosil 2007; García‐Ramos and Kirkpatrick 1997). However, migration load is likely rare in natural populations, as significant dispersal over short timescales is uncommon. And in cases of persistent migration, natural selection typically eliminates maladaptive alleles, achieving a migration–selection balance (Alzate et al. 2017; Bolnick and Nosil 2007).

The limited effects of dispersal observed in populations evolving in isolation, especially for *E. coli* , suggest that the benefits of dispersal may have been overestimated in previous studies (Alzate et al. 2017; Berdahl et al. 2015; Freeland 2020; Garant et al. 2007). The efficacy of dispersal in facilitating adaptation depends on two key factors: the genetic diversity of the migrants and the number of individuals introduced. Immigrants with high genetic variation can substantially enrich the genetic pool of focal populations, explaining why dispersal between heterogeneous environments often supports biodiversity more effectively than dispersal between homogeneous habitats (DeAngelis et al. 2016; Savary et al. 2023). Additionally, introducing pre‐adaptive alleles into changing environments can greatly enhance adaptation (Thompson and Fronhofer 2019; Urban et al. 2012). Besides, the number of individuals in dispersed populations is essential. Dispersal may also introduce maladaptive alleles particularly under random movement. These genetic loads would offset the benefits of the introduced beneficial alleles and even make the net fitness change of the focal population negative. Such negative effects of dispersal will become especially obvious when immigrants are in large numbers (Alzate et al. 2017; Bolnick and Nosil 2007; Thompson and Fronhofer 2019). Specially, in those habitats with small carrying capacity, the arrival of large numbers of immigrants will lead to the collapse of the focal population, even if very few maladaptive genes and many beneficial ones are introduced (Gomulkiewicz et al. 1999). Excessive immigrants may also reduce the genetic diversity of the focal population through homogenization. The large genetic load and the reduction in genetic diversity resulting from high dispersal levels may impede adaptation of populations. Therefore, dispersal plays a dual role and the intermediate level of dispersed populations could usually accelerate adaptation (Bolnick and Nosil 2007; Garant et al. 2007; Gomulkiewicz et al. 1999).

In conclusion, our findings demonstrate that dispersal across thermal gradients significantly enhances abiotic adaptation in numerically inferior populations at higher temperatures. Given the intensification of species interactions due to global warming as one of the major causes of current species extinctions (Johansson 2008), our findings provide a possible means of mitigating interspecific competition and thus slowing the extinction of inferior species under global warming.

## Author Contributions

**Dong‐Hao Zhou:** investigation (equal), writing – original draft (supporting), writing – review and editing (equal). **Shu‐Jun Chang:** data curation (equal), writing – review and editing (supporting). **Nan Chen:** conceptualization (lead), data curation (equal), investigation (equal), writing – original draft (lead), writing – review and editing (equal).

## Funding

This work was supported by Yunnan Provincial Department of Education Science Research Fund Project (2025J0630), National Natural Science Foundation of China (32371687).

## Conflicts of Interest

The authors declare no conflicts of interest.

## Supporting information


**Figure S1:** Schematic illustrations of the experimental evolution. Types and nesting hierarchy of cultures (a) and a sample of dispersal scenarios for certain evolving lines within a block (b).

## Data Availability

Data associated with this study are available at figshare (https://figshare.com/s/bd4ed2d35cc6cc3f9dc7).
