# Supplementary material for: Dispersal Enhances Abiotic Adaptation in Populations Under Interspecific Competition Compared to Isolation
Source: Ecol Evol. 2026 Apr 8;16(4):e73358. doi: 10.1002/ece3.73358 (PMC13058819; doi:10.1002/ece3.73358)

**Figure S1.**  Schematic illustrations of the experimental evolution. Types and nesting hierarchy of cultures (a) and a sample of dispersal scenarios for certain evolving lines within a block (b).


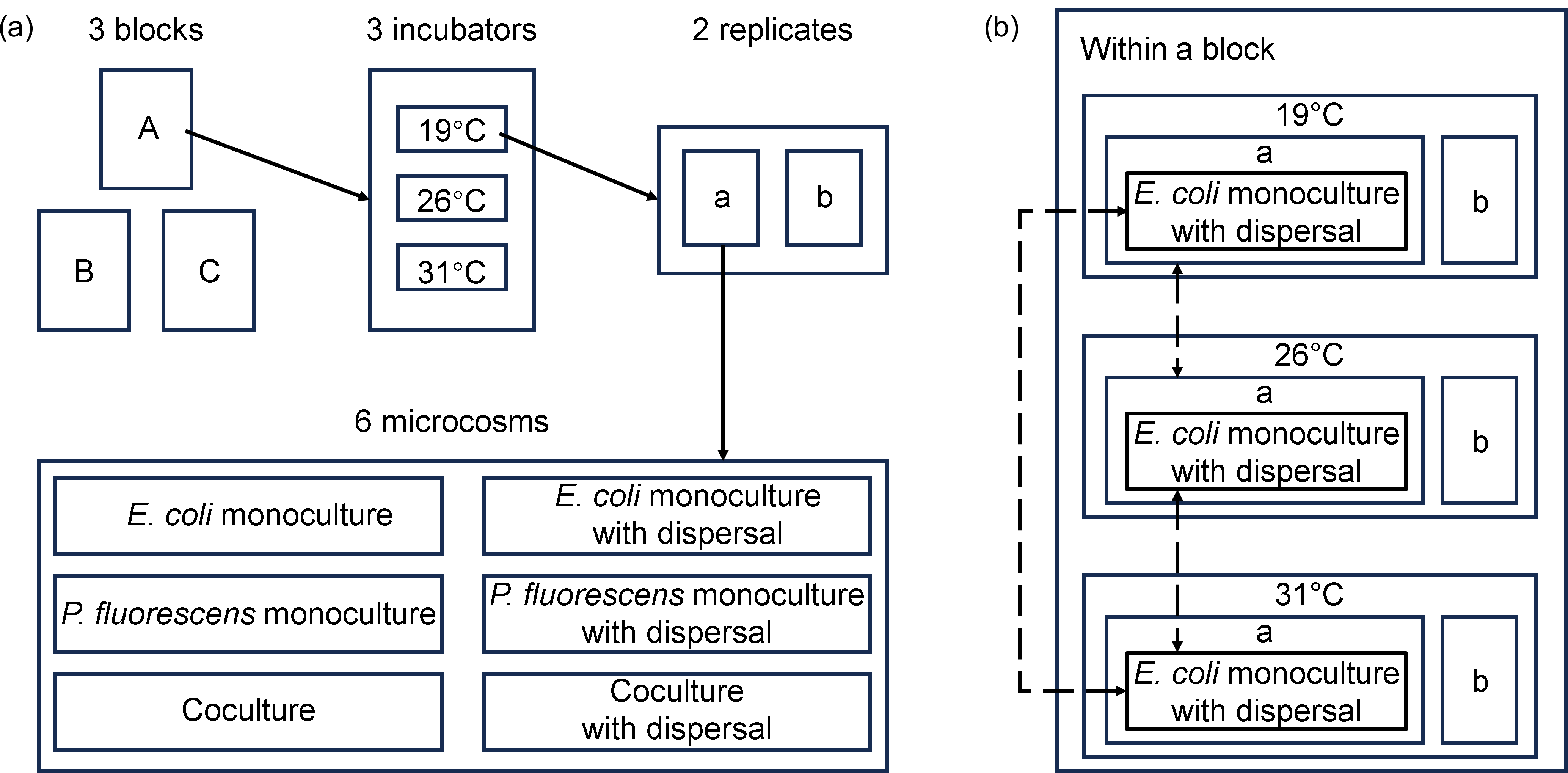

Supplement: Supplementary file 1 — Figure S1: Schematic illustrations of the experimental evolution. Types and nesting hierarchy of cultures (a) and a sample of dispersal scenarios for certain evolving lines within a block (b). [file ECE3-16-e73358-s001.docx]
